# Multimodal-Based Non-Contact High Intraocular Pressure Detection Method

**DOI:** 10.3390/s25144258

**Published:** 2025-07-08

**Authors:** Zibo Lan, Ying Hu, Shuang Yang, Jiayun Ren, He Zhang

**Affiliations:** 1 School of Information Science and Engineering, Shenyang University of Technology, Shenyang 110870, China; 2 Department of Ophthalmology, The Forth People’s Hospital of Shenyang, Huanggu District, No. 20 Huanghenan Street, Shenyang 110031, China; eyehuying@163.com

**Keywords:** non-contact high IOP detection, deep learning, multi-modal model, Scheimpflug imaging

## Abstract

This study proposes a deep learning-based, non-contact method for detecting elevated intraocular pressure (IOP) by integrating Scheimpflug images with corneal biomechanical features. Glaucoma, the leading cause of irreversible blindness worldwide, requires accurate IOP monitoring for early diagnosis and effective treatment. Traditional IOP measurements are often influenced by corneal biomechanical variability, leading to inaccurate readings. To address these limitations, we present a multi-modal framework incorporating CycleGAN for data augmentation, Swin Transformer for visual feature extraction, and the Kolmogorov–Arnold Network (KAN) for efficient fusion of heterogeneous data. KAN approximates complex nonlinear relationships with fewer parameters, making it effective in small-sample scenarios with intricate variable dependencies. A diverse dataset was constructed and augmented to alleviate data scarcity and class imbalance. By combining Scheimpflug imaging with clinical parameters, the model effectively integrates multi-source information to improve high IOP prediction accuracy. Experiments on a real-world private hospital dataset show that the model achieves a diagnostic accuracy of 0.91, outperforming traditional approaches. Grad-CAM visualizations identify critical anatomical regions, such as corneal thickness and anterior chamber depth, that correlate with IOP changes. These findings underscore the role of corneal structure in IOP regulation and suggest new directions for non-invasive, biomechanics-informed IOP screening.

## 1. Introduction

Glaucoma is the leading cause of irreversible blindness globally, affecting over 80 million people. With an aging population and limitations in current screening methods, the number of affected individuals is expected to increase to 111 million by 2040, representing a 38.75% rise [1]. This neurodegenerative disease gradually damages the optic nerve, resulting in progressive loss of peripheral vision and, eventually, complete blindness [2,3]. Epidemiological studies indicate that, even in medically advanced countries, up to 50% of glaucoma cases remain undiagnosed [4]. This highlights the major limitations in current diagnostic methods for early detection and precise monitoring of the disease.

The pathogenesis of glaucoma involves multiple factors, involving age-related changes, genetic factors, and vascular dysregulation [5,6]. Among these factors, IOP is the only clinically modifiable risk factor, as supported by extensive clinical evidence [7,8]. IOP homeostasis relies on the aqueous humor circulation system. The ciliary body continuously produces aqueous humor at a rate of 2.0–3.0 μL/min. This fluid flows through the anterior chamber and is primarily drained through the trabecular meshwork (75–80%) and secondarily through the uveoscleral pathway (20–25%) [9,10]. Maintaining normal IOP requires a precise balance between production and outflow. Any pathological alterations that disrupt this dynamic can lead to increased IOP, inducing mechanical stress on the retinal ganglion cell axons and their corresponding pathways. Research shows that a 10% reduction in IOP corresponds to an approximately 50% decrease in the risk of glaucoma progression [11]. This highlights the critical importance of accurate IOP monitoring in the management of glaucoma.

Currently, Goldmann applanation tonometry (GAT) is still regarded as the gold standard for intraocular pressure (IOP) measurement, but its basic principle has limitations. Firstly, the method relies on the assumption of an idealized corneal model, neglecting the impact of various coupled corneal biomechanical properties on IOP measurement accuracy. Parameters such as corneal stiffness (corneal hysteresis), central corneal thickness (CCT), and curvature can significantly influence pressure readings through biomechanical coupling effects [12]. Clinical evidence suggests that thinner CCT may underestimate true IOP. For instance, when CCT is close to 520 microns, deviations of ±50 microns can result in errors of 1.0–2.5 mmHg, and an error of 1.5 mmHg can have a substantial impact on clinical management [13]. Similarly, abnormal corneal hysteresis or anterior chamber depth can cause distorted pressure readings. Furthermore, axial curvature, and apex shape not only influence IOP readings through biomechanical properties, but may themselves be physiological factors affecting IOP, reflecting the interaction between corneal structure and aqueous dynamics. Secondly, the process of obtaining IOP readings requires applying external force to the cornea, which may alter corneal morphology and aqueous dynamics through mechanical-biological coupling effects, leading to interference in the test system due to the disturbance of the observed system. Non-contact tonometry, such as air-puff methods, avoids direct contact but still induces corneal deformation to estimate IOP, which remains susceptible to individual differences in corneal biomechanics. As a result, both approaches exhibit limitations in accuracy and repeatability across different patient populations.

These inherent limitations in measurement accuracy and repeatability have driven researchers to explore estimation methods that do not rely solely on direct physical interaction with the eye. Traditionally, IOP estimation has employed multivariate regression models to quantify the relationships between clinical variables such as CCT, anterior chamber depth (ACD), and systolic blood pressure with IOP values [14,15,16]. However, these statistical approaches are constrained by their assumption of linearity and struggle to capture the complex nonlinear interactions among ocular and systemic parameters. Moreover, some traditional methods treat corneal structural parameters merely as confounding variables to be adjusted for, rather than recognizing their possible active role in IOP regulation via the aqueous humor–corneal biomechanical feedback system. This simplification potentially overlooks essential biomechanical insights. In recent years, advances in ophthalmic imaging and deep learning have opened new avenues for noninvasive IOP estimation. Notably, deep learning has demonstrated considerable success in image-based disease classification tasks, particularly in glaucoma detection using retinal fundus images [17,18,19]. However, its application to direct, quantitative IOP prediction remains limited [20,21,22]. Unlike conventional statistical models, deep learning approaches are well suited to handling nonlinearities and integrating heterogeneous data sources, including corneal biomechanics and imaging features, offering a promising alternative for more accurate and individualized IOP estimation.

**Figure 1:**
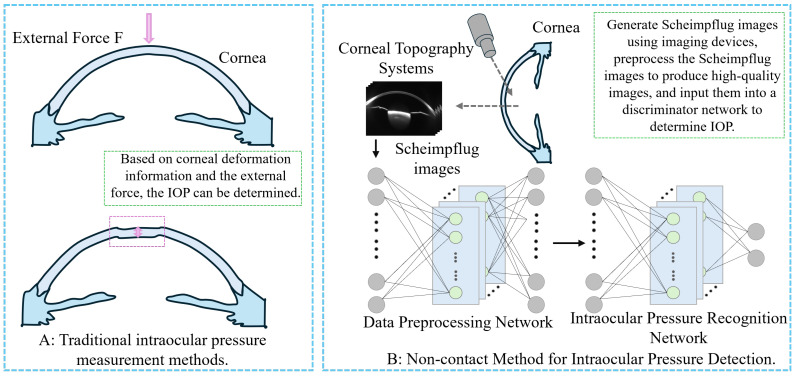
Comparison of IOP measurement methods. (**A**) Schematic diagram of traditional contact-based IOP measurement method. (**B**) Schematic diagram of the non-contact IOP measurement method proposed in this study.

To overcome the limitations of both contact and existing non-contact techniques, this study explores a truly non-contact IOP detection method that eliminates the need for corneal deformation and mechanical force application. This study innovatively proposes a deep learning-based non-contact IOP detection method (Figure 1B). First, Scheimpflug images of the eye and associated ocular parameters were collected. Given the limited quantity of Scheimpflug image data, we applied a Cycle-Consistent Generative Adversarial Network (Cycle-GAN) for data augmentation to expand the size and diversity of the dataset. Simultaneously, a Broad Learning System (BLS) was employed to perform regression discrimination on the generated data, filtering out low-quality images to ensure the high quality of the dataset. To further enhance detection performance, transfer learning was utilized, and all processed data were input into a Swin Transformer model for training. By extracting feature maps from certain layers of the model and incorporating ocular parameter information, these features were subsequently input into the KAN network for further training. Ultimately, accurate detection of high IOP was achieved.

This method minimizes biomechanical interference by avoiding any mechanical contact or induced deformation of the cornea, thus achieving a more physiologically faithful estimation of IOP. This study not only validates existing statistical conclusions but also demonstrates that valuable IOP predictive biomarkers can be extracted from original corneal images using deep learning. The results highlight the pivotal role of corneal structure in IOP regulation and provide new insights for developing precise IOP models that integrate corneal parameters.

## 2. Related Work

This chapter provides a brief review of the fundamental principles and clinical limitations of current mainstream IOP measurement technologies, with a focus on key corneal biomechanical factors that affect measurement accuracy. Subsequently, the chapter further summarizes the research progress and inherent limitations of physical model-based correction methods.

### 2.1. Technical Principles and Clinical Limitations of Intraocular Pressure Measurement Devices

IOP measurement is a critical parameter in the diagnosis and management of glaucoma, as its accuracy directly impacts disease staging and treatment decisions. Although current mainstream clinical devices are based on different physical principles, they generally rely on standardized assumptions about corneal biomechanics. This common characteristic leads to measurement deviations under certain corneal conditions, which require further in-depth analysis.

#### 2.1.1. Contact Tonometry Technology

The Schiotz applanation tonometer, based on the mechanical contact principle [23], estimates IOP by measuring corneal indentation depth. Its physical model assumes that the cornea behaves as an ideal, homogeneous elastic material. However, recent biomechanical studies show that the cornea’s viscoelastic properties significantly affect indentation dynamics [24], leading to systematic measurement biases in clinical applications. This highlights the limitations of the traditional mechanical model in analyzing dynamic responses.

Applanation tonometry is still regarded as the most reliable method for accurate IOP measurement. Among these methods, the Goldmann Applanation Tonometer (GAT) is internationally recognized as the clinical gold standard. Its underlying principle is based on an idealized model derived from Imbert-Fick’s law. The device exerts vertical pressure using a flat-headed probe with a 60° cone angle. When a circular flattened area with a diameter of 3.06 mm is formed at the corneal center, the IOP can be expressed as shown in Equation (1):

(1) IOP=F/A

The force (*F*) applied to the eye and the contact area (*A*) between the probe and the cornea are critical factors in applanation. The force required to flatten the corneal surface is directly proportional to IOP. This classical equation is based on two key assumptions: (1) the cornea behaves as a perfectly elastic membrane with uniform thickness (typically 520 μm); (2) the corneal curvature radius remains constant at 7.8 mm. However, in clinical reality, the cornea exhibits non-ideal properties such as anisotropy, viscoelasticity, and regional biomechanical variation [25]. These factors alter the force required for applanation independently of IOP, introducing significant measurement errors [26,27]. For instance, a stiffer cornea tends to yield higher IOP readings, while a more compliant cornea results in underestimation. Clinical studies have shown that a 10% variation in CCT can lead to changes in IOP measurements of up to 3.4 ± 0.9 mmHg (p≤ 0.001, *r* = 0.419) [28,29]. Similarly, variations in corneal curvature and viscoelastic response further affect the accuracy of GAT readings, particularly in post-surgical or pathological corneas [30,31]. These biomechanical limitations highlight the need for tonometry techniques that incorporate individualized corneal properties.

#### 2.1.2. Non-Contact Tonometry

The non-contact tonometer (NCT) was first introduced in 1972 [32]. Over the past decades, various types of air-puff tonometers have been developed. These devices use an air pulse to flatten the cornea without direct contact, effectively eliminating the risk of cross-infection associated with contact-based measurements. Additionally, they do not require anesthesia or fluorescein instillation, making them more acceptable for certain patient populations. NCT devices achieve momentary corneal flattening primarily through high-speed air pulses [33]. It is worth noting that manufacturers of NCT devices typically treat their core algorithms as trade secrets. However, the fundamental preprocessing algorithms can be traced back to Equation (2):

(2) $$w=d\cdot We/(K_{1}+K_{2}\cdot Bo)$$

Bo represents a composite coefficient that describes the effects of gravity, surface tension, and inertia. Similarly, the Weber number (We) is a coefficient that compares the influences of surface tension and inertia. Here, *d* is 50 μm, and $K_{1}$ and $K_{2}$ are scaling parameters. The specific values of these coefficients can be found in the reference [33].

However, dynamic corneal response analysis has shown that corneal viscoelasticity is closely related to IOP measurement, supporting the hypothesis that viscous resistance may influence tonometer readings [34]. Similarly, in reference [35], the authors propose a viscoelastic model explaining how both viscosity and elasticity affect corneal hysteresis, which is particularly important in IOP measurement. Hysteresis reflects the pressure differences in the cornea during loading and unloading, potentially leading to inaccuracies in tonometer measurements.

#### 2.1.3. Corvis ST

Dynamic response tonometry, represented by Corvis ST, introduced a paradigm shift from static IOP measurement to biomechanical dynamic characterization [36]. Using high-speed Scheimpflug imaging, Corvis ST captures corneal deformation under an air pulse. The system records parameters like applanation times (A1 and A2), maximum deformation amplitude (DA), and deformation velocity (SP-HC) to construct a biomechanical response curve, such as a stress–strain hysteresis loop. Notably, the biomechanically corrected IOP (bIOP) algorithm in Corvis ST incorporates the corneal stiffness parameter (SP-A1) for the first time in IOP calculation. This approach theoretically reduces the influence of corneal thickness variability on measurement accuracy.

However, Corvis ST’s core algorithm has three main limitations. First, the biomechanical model assumes population-averaged viscoelastic parameters, which may overlook individual differences. Second, the nonlinear relationship between dynamic response parameters and true IOP relies on empirical regression models, which can affect measurement precision. Lastly, the system reduces multidimensional biomechanical parameters to a single correction factor for bIOP, which may fail to fully account for interactions between corneal properties.

### 2.2. Biomechanical Interfering Factors in IOP Measurement

The basic principle of IOP measurement relies on applying a standardized mechanical force to the eyeball. By continuously monitoring corneal deformation (such as flattening rate, rebound acceleration, and other dynamic parameters), IOP is calculated through inverse modeling in conjunction with a biomechanical model. It is clear from this principle that the measurement results of existing clinical technologies are inevitably influenced by corneal tissue parameters. Notably, the contribution of different corneal parameters to IOP measurements varies significantly, primarily due to the complexity of the cornea’s anisotropic tissue structure.

#### 2.2.1. Structural Parameter Dependency

CCT refers to the thickness of the central part of the cornea and is considered a key indicator of its biological characteristics. The cornea, as the transparent outer layer of the eye, protects the eyeball, focuses light, and contributes to refractive functions. CCT is an important biomechanical parameter of the cornea. Corneal thickness can influence the cornea’s elasticity, stiffness, and its response to external pressure. A thinner cornea may indicate reduced elasticity, while a thicker cornea could suggest increased stiffness [37].

Corneal Radius of Curvature refers to the distance from the corneal surface to its center, representing the radius of the circle formed by this distance. In simple terms, it describes the degree of corneal curvature: flatter corneas have a larger radius, while steeper corneas have a smaller radius. Common IOP measurement techniques, such as non-contact tonometry and GAT, depend on the corneal shape. Therefore, corneal curvature directly affects the accuracy of the measured values.

#### 2.2.2. Dynamic Response Characteristics

Corneal Hysteresis (CH) refers to the cornea’s ability to respond to changes in IOP, acting as a buffer against pressure fluctuations. It reflects the cornea’s biomechanical properties, including elasticity and compliance. Tonometry measurements of IOP are affected by corneal elasticity. Assessing corneal hysteresis provides a more accurate evaluation of an individual’s IOP status [38,39].

#### 2.2.3. Influence of Microstructure

The corneal stroma is the main component of the cornea, constituting about 90% of its structure. It consists of water, collagen fibers, and glycosaminoglycans, with collagen fibers being the most important, providing transparency, strength, and elasticity to the cornea. Collagen fibers in the corneal stroma are organized, not randomly arranged. Previous studies show that the orientation of collagen fibers in the corneal stroma regulates IOP. Other studies have found that abnormal distribution of these fibers can alter corneal shape, affecting IOP measurements [40,41].

### 2.3. Physical Model Correction for IOP Measurement

The core principle of IOP measurement technology is to apply external force to the cornea and estimate IOP based on corneal rebound values. However, corneal biological parameters are heterogeneous and exhibit nonlinear interactions, which cause inaccuracies in the standardized models used by current IOP measurement methods. These inaccuracies lead to discrepancies in the predictive performance of theoretical models, reducing their reliability in clinical applications. Consequently, some researchers have focused on improving the physical models used for IOP measurement.

The Goldmann Applanation Tonometer measures IOP based on the Imbert-Fick law, but it overlooks many structural details of the cornea, leading to discrepancies in the measurements. To address this issue, research has been conducted since 1975 [42]. In these studies, researchers applied both single and multiple linear regression models to analyze the data and evaluated the significance of the results using the t-distribution. The Kolmogorov–Smirnov test also confirmed that the data followed a Gaussian distribution, meeting the prerequisites for such statistical analyses. The final Ehlers correction formula is shown in Equation (3), where IOPG represents the Goldmann measured result:

(3) IOP=IOPG−[5.0×(((CCT/1000)−0.520)/0.070)]

Similarly, some researchers have used mathematical modeling to derive Goldmann’s actual measurement results [43]. They modeled the cornea as a thin shell and applied deformation theory to describe its response to intraocular and measurement pressures. Based on this, they derived the relationship between true IOP, corneal dimensions, and measured IOP as shown in Equation (4):

(4) IOP=IOPG/K

*K* is a correction factor that depends on corneal dimensions, radius, and thickness.

Clinical studies have also shown that corneal characteristics affect the accuracy of Goldmann measurements [44]. To investigate this, researchers examined 125 eyes. They measured CCT using an ultrasound pachymeter, assessed IOP with a Perkins tonometer after anesthesia, and derived an IOP correction formula using statistical methods, as given in Equation (5):

(5) IOP=IOPG+(23.28−0.0423×CCT)

Given that the linear model for correcting IOP measurements is not precise, Elsheikh et al. [45] used nonlinear finite element simulations, considering additional corneal parameters such as central CCT, central anterior curvature, age, and their effects on IOP. They proposed a multiparameter correction equation, as given in Equation (6):

(6) $$C=\frac{IOPG}{IOP}=A_{CCT}\cdot {\left(CCT-0.520\right)}^{2}+A_{R}\cdot \left(R-7.8\right)+A_{Age}\cdot \left(Age\right)+A_{IOPG}$$

*C* is a correction factor used to adjust the intraocular pressure (IOPG) measured by the Goldmann tonometer, bringing it closer to the true IOP value. $A_{\mathrm{CCT}},A_{R},A_{\mathrm{Age}},A_{\mathrm{IOPG}}$ are constants derived through parameter optimization, representing the contribution of each parameter to the correction factor. The specific values can be found in the literature [45].

### 2.4. Problem Statement

Although these correction models have reduced measurement bias to some extent, their general applicability remains limited by two fundamental challenges. First, traditional linear correction models inherently fail to capture the biomechanical properties of the cornea [46], while previous studies have introduced nonlinear assumptions to describe the dynamic coupling effects between corneal parameters, a second issue still hinders their practical application. Specifically, corneal parameters vary significantly among individuals in clinical practice, making it difficult to accurately account for individual differences using population-based statistical averages. As a result, these models do not fully meet the precision requirements for medical measurements.

To overcome the limitations of linear models, researchers have started exploring the use of machine learning for IOP assessment. For example, algorithms like random forests have been applied in long-term IOP monitoring studies [47,48], showing better performance than traditional statistical models. However, it is important to note that these methods are essentially extensions of the statistical analysis paradigm. They mainly establish data associations using nonlinear algorithms but have not fully explored the deep spatial correlations among biomechanical parameters.

Deep learning techniques, particularly Convolutional Neural Networks (CNNs), provide new approaches to addressing these challenges. Compared to traditional statistical models and machine learning methods, CNN have distinct architectural advantages. First, CNN use multilayer nonlinear transformations to extract morphological associations in high-dimensional feature spaces. This enables them to identify corneal imaging biomarkers that conventional methods cannot detect. Second, their end-to-end training approach enables the rapid establishment of complex mapping relationships between input data and target variables. This forms a technical foundation for IOP prediction models based on corneal features. With advances in hardware and the maturation of CNN technology, these networks have shown substantial clinical value in ophthalmic image analysis and disease screening.

Scheimpflug images, as a crucial tool in clinical diagnosis, contain multidimensional biomechanical information, such as curvature distribution and corneal thickness gradients, which exhibit significant spatial heterogeneity. These features may contain biomarkers closely related to IOP changes. Using CNN to analyze Scheimpflug images can break through the linear assumptions of traditional statistical models. By leveraging a hierarchical feature extraction mechanism, it enables the capture of biomechanical associations between biomarkers and IOP. Clinical information from the eye can partially reflect the IOP value. Through multimodal integration, nonlinear coupling analysis of multiple corneal parameters can be achieved. Furthermore, analyzing multiple parameters offers higher robustness against individual heterogeneity, making it possible to establish personalized IOP prediction models. This provides new insights for non-contact IOP measurement.

## 3. Dataset Construction

In this study, the construction of a high-quality, multimodal dataset was fundamental to ensure the accuracy, generalizability, and clinical relevance of the proposed deep learning framework. Considering the specialized nature of Scheimpflug images and the challenges associated with data scarcity, especially for pathological cases such as high IOP, we adopted a rigorous methodology encompassing data acquisition, augmentation, quality control, and splitting strategies. This chapter details the source and composition of the dataset, the techniques employed to address class imbalance and limited sample size, the implementation of quality control mechanisms to filter low-quality synthetic data, and the specific protocols used to divide the dataset for model training and evaluation.

### 3.1. Data Source and Description

This study utilized a private dataset provided by the Ophthalmology Center of the Fourth People’s Hospital of Shenyang. The study was approved by the Ethics Committee of the Fourth People’s Hospital of Shenyang (approval number: 20250507001, approval date: 7 May 2025). As the dataset contains sensitive patient information and is reserved for future research, it is not publicly available. The dataset comprises multimodal corneal imaging data, including Scheimpflug image samples from 780 eyes of 417 patients, along with corresponding clinical numerical information for eight attributes: Age, pupil center thickness, corneal vertex thickness, corneal volume, anterior chamber depth, anterior chamber volume, corneal diameter, and IOP. All clinical measurements were performed by three associate chief physicians, each with over five years of clinical experience. Each measurement was repeated three times, and the average value was used as the final result. The data were categorized into two groups based on IOP levels: the normal group (IOP ≤21 mmHg), which included 680 samples (87.2%), and the high IOP group (IOP > 21 mmHg), which included 100 samples (12.8%). Detailed clinical information of the dataset is presented in Table 1.

### 3.2. Data Augmentation and Intended Purposes

This study proposes a deep network framework with a two-stage training process. First, since the pre-trained weights of the Swin Transformer are derived from the ImageNet image dataset, which significantly differs in structure and distribution characteristics from Scheimpflug imaging data, transfer learning is required for network adaptation. The goal of this transfer learning phase is not to directly perform classification tasks but to enable the Swin Transformer network to extract structural features from Scheimpflug images. Therefore, the synthetic images used do not need to possess high clinical realism but should emphasize the diversity and generalization of the structural feature space. Upon completion of this training phase, the resulting model is used as a feature extractor for the final training of the KAN. The KAN training is based on real Scheimpflug images and their corresponding clinical annotations, aiming to identify and characterize key anatomical features of the cornea and anterior chamber structures.

Given that Scheimpflug imaging devices are expensive and complex to operate, only 780 valid images were collected in this study, with high IOP cases constituting only 12.8% of the dataset. To address the problem of insufficient samples, especially in the high IOP category where data is severely scarce, a Cycle-GAN was introduced for image-level data augmentation [49]. Unlike traditional augmentation techniques such as rotation, scaling, and mirroring, Cycle-GAN can learn the underlying distribution mapping between different Scheimpflug image domains. This allows it to generate synthetic images with clinically meaningful variations (e.g., differences in corneal thickness and anterior chamber depth), thereby enhancing image diversity and the expression of pathological features.

In the Cycle-GAN framework, the generator simulates natural variations of corneal and anterior chamber structures under clinical conditions, producing image samples with representative structural differences. The discriminator learns to distinguish between real and synthetic images, driving the generator to continuously improve image quality. The introduction of cycle consistency loss ensures that the generated images can be reversibly mapped back to the original images, further enhancing the stability of the training process and the controllability of the generated results. In this study, a total of 1200 augmented images (600 normal IOP and 600 high IOP images) were synthesized and used in the transfer learning phase of the Swin Transformer module to enhance its structural feature perception capability. However, these images were not used in the final training phase of KAN to avoid any potential biases in clinical realism that could negatively affect predictive performance.

**Figure 2:**
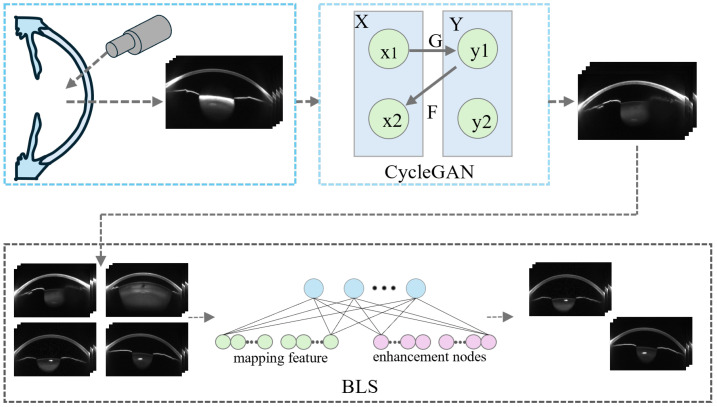
Data Augmentation Framework.

Although Cycle-GAN has strong image generation capabilities, it lacks precise control, which may lead to severe structural deviations in the synthetic images. These deviations could result in the model learning non-existent structural information. To address this issue, we further introduce a BLS to filter the structural quality of the synthetic images. This ensures that the samples used in the transfer learning phase meet the requirements for structural validity and discriminative information, thereby enhancing the stability and generalization of the transfer learning process. The data augmentation framework is illustrated in Figure 2.

### 3.3. Data Quality Control Strategy

Cycle-GAN lacks quality monitoring capabilities, which can result in significant structural issues in the synthetic data generated by GANs, such as corneal boundary artifacts, unrealistic lens structures, and excessive random noise. Therefore, a quality monitoring module is necessary to filter the synthetic data. Traditional CNNs require large labeled datasets and substantial computational resources, making them unsuitable for this study. The limited availability of real data and the severe imbalance between high IOP and normal IOP samples (100:680) makes it difficult to train an effective quality monitoring model using this data alone. For this reason, we chose BLS as the quality monitoring model [50].

Unlike the backpropagation algorithm, BLS primarily relies on pseudoinverse matrix calculations to solve the output weights during training. This approach allows the model to update parameters in a single iteration, significantly improving training speed. Additionally, BLS supports an incremental learning mechanism, which enables the dynamic addition of new feature or enhancement nodes to improve model performance without retraining the entire model. This feature makes it highly suitable for the current research scenario. As the amount of data generated by Cycle-GAN increases, BLS can quickly update the network parameters through pseudoinverse matrix calculations, enhancing its ability to monitor the quality of synthetic data. Previous studies have shown that BLS can effectively serve as a quality control system, demonstrating its ability to handle high-dimensional data and provide robust results even with limited training data [51].

BLS is an incremental learning model derived from the Random Vector Functional Link Network (RVFLN). Its core principle is to achieve efficient classification by cascading feature and enhancement nodes within a single-layer architecture. Given an input sample set $X=\{x_{1},x_{2},\ldots ,x_{n}\}$, a randomly initialized weight matrix $W\in R^{d\times m}$ and bias vector $b\in R^{m}$ are employed to generate the feature nodes via a nonlinear mapping, h=σ(WX+b), where σ denotes an activation function, *d* is the input dimension, *m* is the number of feature nodes, and *n* is the number of samples. The feature nodes *h* are further transformed into enhancement nodes using a second nonlinear mapping, $z=\sigma (W_{e}h+b_{e})$), where $W_{e}\in R^{m\times p}$ and $b_{e}\in R^{p}$ represent the weights and biases of the enhancement layer, respectively, and *p* denotes the number of enhancement nodes. This transformation enhances the network’s capacity to capture complex nonlinear relationships in the data.

During the classification decision phase, the BLS model determines the category of each sample by comparing the generated regression score with a predefined threshold *T* = 0.7. Specifically, the model computes a similarity measure between the features of the generated image and those of the corresponding real image. This similarity is quantified as a regression score, which serves as an indicator of the generated image’s quality. The threshold value of 0.7 is empirically determined based on experimental results and observations of the data distribution, aiming to effectively distinguish between high-quality and low-quality image samples. If the regression score is greater than or equal to 0.7, the sample is classified as high-quality. otherwise, it is considered low-quality. This threshold-based mechanism provides a robust and interpretable criterion that enables the BLS model to reliably monitor the quality of synthesized images and make informed classification decisions accordingly. The classification decision is made according to the following Equation (7):

(7) $$f\left(z\right)=w^{T}z+b$$

If f(z)≥T, the sample is classified as high-quality; f(z)<T, the sample is classified as low-quality.

In the implementation, the BLS network consists of 1000 feature nodes, which extract features through random mapping. The feature extraction process can be expressed by Equation (8):

(8) $$h_{i}=\sigma \left(W_{i}x_{i}+b_{i}\right),\;i=1,2,\ldots ,1000$$

where $W_{i}\in R^{d\times 1}$ is the weight vector of the *i*-th node, $b_{i}$ is the bias term, and $x_{i}$ is the input of the *i*-th sample. Simultaneously, 600 enhancement nodes are set in the BLS, using a sigmoid activation function for enhancement. The output of the enhancement node layer is calculated as shown in Equation (9):

(9) $$z_{i}=\sigma \left(W_{e}h_{i}+b_{e}\right),\;i=1,2,\ldots ,600$$

Finally, ridge regression is used to compute the regression score. The regression score from the output layer is calculated by the following formula (Equation (10)):

(10) $$f\left(z\right)={\left(Z^{T}Z+\lambda I\right)}^{-1}Z^{T}y$$

where *Z* is the output from the enhancement nodes, *y* is the corresponding regression label, and λ is the regularization parameter.

The incremental learning capability of BLS allows it to dynamically adjust the classification model for different batches of generated data. If initial performance is unsatisfactory, its discriminative ability can be improved by incrementally adding enhancement nodes—without requiring complete retraining.

### 3.4. Data Splitting

In accordance with the gold standard for medical image analysis, we employed a stratified sampling strategy to divide the dataset, ensuring that: (1) the ratio of normal to high IOP images matches the original distribution, and (2) multiple images from the same patient appear in only one subset.

The model training involved two phases: one for transfer learning and another for final training with medical numerical data. First, we conducted transfer learning using 1200 generated Scheimpflug images over 50 epochs. Then, we trained the KAN model for 100 epochs using 780 real-world images and their corresponding medical numerical data. We divided the dataset into training, validation, and test sets using an 8:1:1 ratio. Specifically, the dataset was split into a training set (624 images, 80%) for parameter optimization, a validation set (78 images, 10%) for monitoring overfitting and hyperparameter tuning, and a test set (78 images, 10%) solely for performance evaluation. Notably, all samples underwent DICOM anonymization.

## 4. Proposed Method

**Figure 3:**
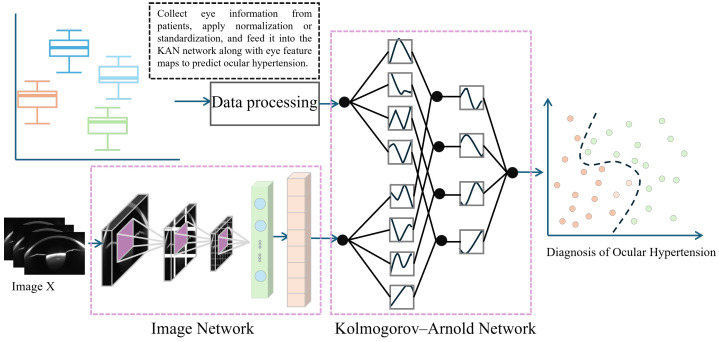
Multimodal IOP Measurement Framework.

The input data is first fed into the Swin Transformer model [52], which leverages its powerful image feature extraction capabilities to generate multi-level feature maps. These feature maps, along with eye parameter information, are then fed into the KAN network for further training and optimization. Ultimately, the trained model accurately identifies high IOP cases, providing effective decision support for clinical practice. The complete framework is shown in Figure 3.

### 4.1. Image Feature Extraction

Accurate feature extraction is a critical prerequisite for successful image-based diagnosis in medical imaging tasks. In the context of Scheimpflug image analysis, the complexity of ocular structures and the indirect nature of IOP indicators present significant challenges for conventional models. To overcome these limitations, we adopt a self-attention-based architecture as the feature extractor, which is well suited for capturing both local and global contextual information across multiple ocular regions.

#### 4.1.1. Network Backbone Architecture

CNNs have been widely applied in medical image analysis, particularly in ophthalmology, for tasks such as detecting various eye conditions. However, high IOP detection presents unique challenges. Unlike other eye diseases, IOP is indirectly related to factors like corneal thickness, and its measurement is influenced by multiple regions of the eye, rather than any single factor or area. This complexity makes it difficult for traditional CNN models, which focus on local features, to accurately capture the intricate, multiregional relationships involved in IOP detection.

To address these challenges, this study employs the Swin Transformer, which offers a more flexible and efficient approach for processing medical images. In particular, the Swin Transformer’s local window-based self-attention mechanism enables the model to capture both local and global features across the image. This capability allows the model to consider complex, multi-regional dependencies, making it especially effective for IOP detection, where subtle relationships must be integrated for accurate diagnosis. Moreover, the Swin Transformer introduces a key advantage over other Transformer-based models, such as the Vision Transformer (ViT) [53], while both ViT and Swin Transformer are built on similar Transformer principles, the Swin Transformer incorporates a hierarchical design that progressively enlarges the receptive field through windowed attention at each layer. This structure not only improves efficiency in capturing multiscale features but also enhances the model’s ability to process long-range dependencies. In contrast, ViT models typically use a fixed attention window, which does not scale as effectively across varying levels of detail in the image. As a result, the Swin Transformer is better equipped to handle the complexity and subtlety of high IOP detection, where fine-grained, multiscale features play a crucial role.

**Figure 4:**
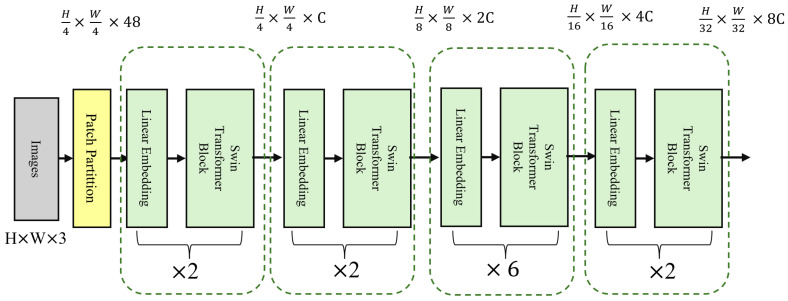
Main Architecture of the Swin Transformer Model.

The Swin Transformer consists of several core modules (Figure 4), each containing three key components: Patch Partition, Window-based Multi-head Self Attention, and Shifted Window Mechanism. First, Patch Partition divides the input image into fixed-size patches, which are then linearly mapped into feature vectors for further processing. The Swin Transformer then performs self-attention calculations within each local window to capture local features. The Shifted Window Mechanism shifts the windows progressively at each layer, facilitating interactions between different windows and enhancing the model’s ability to capture global information. This mechanism helps the model balance local details and global structure, significantly improving high IOP detection performance.

In this study, we used the standard Swin Transformer configuration. The input image size was 224 × 224 pixels, and we applied a 4 × 4 patch size for downsampling to divide the image into smaller patches. In the self-attention module, we employed a multi-head attention mechanism. The model consists of four stages, with configurations of (2, 2, 6, 2), and the number of attention heads in each stage was (3, 6, 12, 24), respectively. In each layer, we performed two main operations: window-based self-attention computation and window shifting. After each layer, we normalized the output using layer normalization to maintain the model’s stability.

To improve training efficiency and prevent overfitting, we adopted transfer learning by fine-tuning a Swin Transformer model pre-trained on ImageNet. During training, we used the cross-entropy loss function and the AdamW optimizer. We also applied learning rate decay during the initial stages to accelerate model convergence.

#### 4.1.2. Transfer Learning for High IOP Detection

We selected the Swin Transformer model as the backbone network because it has been pre-trained on the ImageNet dataset [54], which contains a rich set of visual information. This allows the model to learn general visual features such as edges, textures, and shapes. These features are essential in most image analysis tasks and form a strong foundation for our high IOP detection task. However, since high IOP detection mainly relies on structural features in Scheimpflug ophthalmic images, a model trained solely on ImageNet may not be fully suited for this specific task. Therefore, we fine-tuned the model using augmented Scheimpflug image data to better adapt it to the characteristics of ophthalmic images.

During the transfer learning process, we employed an adaptive fine-tuning strategy. We froze certain layers of the model while fine-tuning the weights of others. Specifically, we froze the lower convolutional layers and the shallower Transformer layers, which extract general low-level features (e.g., edges, textures) that are common across many types of images. Since these features are widely applicable, we did not update the weights of these layers. For the higher-level modules, especially those related to task-specific feature extraction, we unfroze and fine-tuned them. This approach allowed us to focus the model more effectively on the specific task, improving the accuracy of high IOP detection.

#### 4.1.3. Hierarchical Feature Selection

**Figure 5:**
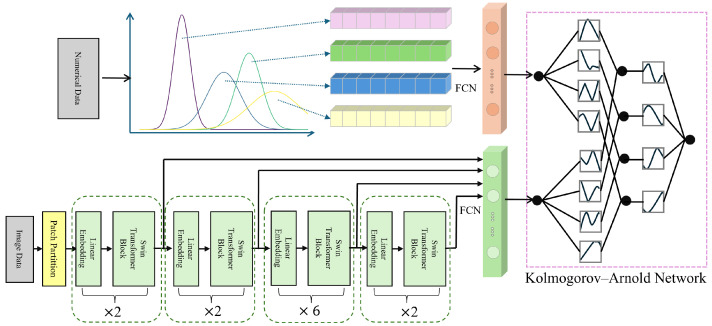
Schematic Diagram of Fusion of Numerical Clinical Information and Feature Maps.

When performing high IOP detection, the Swin Transformer extracts rich feature information through its hierarchical network structure. These features can be classified into low-level and high-level features, corresponding to the basic structure and more complex semantic information of the image, respectively. During feature extraction, low-level features (such as edges and textures) capture fine details of ophthalmic images, while high-level features contribute to medical interpretation. To improve high IOP detection accuracy, we combined features from each thematic module of the model. By applying feature selection and fusion strategies, we extracted the most diagnostically valuable features and used them as input for the KAN model (Figure 5).

### 4.2. Feature Fusion of Imaging and Clinical Data

In medical image analysis, ophthalmic images and clinical parameters provide complementary information that is crucial for disease diagnosis. However, traditional image classification methods generally focus on image data alone, neglecting the valuable contributions of clinical parameters. To address this limitation, this study employs the Kolmogorov–Arnold Net (KAN) [55]. The KAN network integrates both image and clinical data to enhance the accuracy of high IOP detection.

#### 4.2.1. KAN-Based Multimodal Fusion

KAN is a novel neural network architecture proposed in 2024, distinguished by its use of learnable one-dimensional spline functions on edges instead of fixed activation functions on nodes, as in conventional multilayer perceptrons (MLPs). This edge-wise design removes the need for linear weight matrices, allowing each node to perform purely additive operations. As a result, the model gains enhanced function approximation capabilities and interpretability. Each edge can be visualized as a function curve, providing intuitive insight into the model’s decision-making process.

Unlike conventional multimodal fusion strategies—such as simple feature concatenation—that fail to explicitly model inter-modal relationships, KAN leverages nonlinear function decomposition to integrate ophthalmic images and clinical parameters within a shared latent space. This enables it to capture subject-specific, nonlinear interactions between modalities, such as those between corneal thickness, IOP, and image-derived features. Such modeling is particularly beneficial for high-IOP detection tasks, leading to improved predictive performance and more robust multimodal representation learning.

#### 4.2.2. Parametric Architecture Design

The core design concept of the KAN network is based on the Kolmogorov–Arnold representation theorem, which states that any multivariate nonlinear function can be approximated by a finite set of nonlinear basis functions [56,57]. KAN maps multimodal data into a unified feature space, using a set of nonlinear basis functions to represent the complex relationships within the data. This process can be expressed mathematically as follows (Equation (11)):

(11) $$f\left(x_{1},x_{2},...,x_{n}\right)=\sum_{i=1}^{N}\lambda_{i}\;\phi_{i}\left(x_{i}\right)$$

Here, *f* represents the objective function, $x_{i}$ denotes the input features from different modalities, $\lambda_{i}$ is the weight of each basis function, and $\phi_{i}\left(x_{i}\right)$ is the nonlinear basis function associated with the input modality.

In this study, ophthalmic image data and clinical parameter data are mapped using B-splines. B-spline basis functions are smooth functions with local support, which are particularly suitable for capturing local features (such as subtle morphological changes in CCT) in medical image analysis. Therefore, for processing ophthalmic images, we employed B-spline basis functions (Equation (12)):

(12) $$\phi_{i}\left(x_{i}\right)=\sum_{k=0}^{K}\alpha_{ik}B_{k}\left(x_{i}\right)$$

Here, $B_{k}\left(x_{i}\right)$ represents the *k*-th B-spline basis function, $\alpha_{ik}$ is the corresponding weight, and *k* denotes the number of basis functions. Additionally, to address some global nonlinear relationships, we also used polynomial basis functions for mapping (Equation (13)):

(13) $$\phi_{i}\left(x_{i}\right)=\sum_{m=0}^{M}\beta_{im}x_{i}^{m}$$

where *M* is the degree of the polynomial, and $\beta_{im}$ are the polynomial coefficients. By selecting appropriate basis functions, the KAN network can effectively capture the nonlinear relationships in both ophthalmic images and clinical data.

To further enhance the accuracy of the KAN network in handling complex data, we introduced an adaptive grid refinement strategy. In this strategy, key regions of the image (such as the cornea, anterior chamber, etc.) are assigned higher grid densities, allowing for more precise computations in these areas. Let the input image be denoted as *I*, and its position after grid division is $g_{i}$. The degree of grid refinement is dynamically adjusted using the following function (Equation (14)):

(14) $$g_{i}^{*}=\arg\min_{g_{i}}{∥\nabla I\left(g_{i}\right)∥}^{2}$$

Here, $\nabla I(g_{i})$ represents the gradient of the image at position $g_{i}$, and $∥\nabla I\left(g_{i}\right){∥}^{2}$ measures the degree of variation in that region. Regions with larger gradients are assigned higher computational precision.

#### 4.2.3. Multimodal Input Encoding

In high IOP detection, ophthalmic images and clinical parameters provide complementary information. Image data can reveal morphological changes in the eye, while clinical parameters offer physiological characteristics related to the eye. To effectively integrate this diverse information, the KAN network ensures that each modality contributes to the final decision through the following encoding method.

For ophthalmic images, we use the Swin Transformer to extract feature maps. Then, the extracted feature maps are transformed into a fixed-size feature vector using the Global Average Pooling (GAP) method (Equation (15)):

(15) $$f_{\mathrm{image}}=\frac{1}{H\times W}\sum_{h=1}^{H}\sum_{w=1}^{W}F\left(h,w\right)$$

Here, *H* and *W* represent the height and width of the image, respectively, and F(h,w) is the feature value at position (h,w), which is used to compute the global feature representation of the image.

For the six clinical parameter data $x_{i}$, standardization is applied to transform the data from different modalities into the same scale (Equation (16)):

(16) $$x_{\mathrm{clinical}}=\frac{x_{i}-\mu_{i}}{\sigma_{i}},\;i=1,\ldots ,6$$

Here, μ and σ represent the mean and standard deviation of the clinical data, respectively. The standardized data is then fused with the image features using a weighted embedding strategy. By learning a weight vector w, the model can automatically determine the contribution of each modality (Equation (17)):

(17) $$f_{\mathrm{final}}=w_{\mathrm{image}}f_{\mathrm{image}}+w_{\mathrm{clinical}}x_{\mathrm{clinical}}$$

Here, $w_{image}$ and $w_{clinical}$ are the weights for the image and clinical data, respectively. The model dynamically adjusts these weights based on feedback during the training process to assign higher weights to the most important modality for the high IOP detection task.

Finally, the standardized clinical numerical data is processed through fully connected layers separately from the image features to obtain two-dimensional features, which are then fed into the KAN network (Figure 5).

#### 4.2.4. Regularization Strategies

To prevent overfitting and enhance the model’s generalization ability, the KAN network incorporates two regularization strategies: L1 regularization [58] and a dynamic dropout mechanism [59].

L1 regularization promotes sparsity in feature selection, thereby reducing the interference of irrelevant features with the model. Specifically, the L1 regularization term is added to the loss function (Equation (18)):

(18) $$L_{\mathrm{total}}=L_{\mathrm{task}}+\lambda \sum_{i}\left|w_{i}\right|$$

Here, $L_{total}$ is the task-specific loss function (e.g., cross-entropy loss), λ is the regularization coefficient, $w_{i}$ represents the model’s weight parameters, and $|w_{i}|$ is the L1 norm, which encourages the model to automatically select the most relevant features.

To further prevent overfitting, we employ a dynamic dropout strategy. Unlike the traditional fixed dropout rate, dynamic dropout automatically adjusts the dropout probability p(t) based on feedback during the training process. This probability gradually decreases as training progresses, helping the model maintain more learning capacity in the early stages while regularizing it in the later stages (Equation (19)):

(19) $$p\left(t\right)=p_{0}/\left(1+\beta t\right)$$

Here, $p_{0}$ represents the initial dropout rate, β is the parameter controlling the decay rate, and *t* denotes the current training step. Finally, we employed an early stopping mechanism, which activates when the model’s validation accuracy remains unchanged for 10 consecutive iterations.

## 5. Results and Discussion

This chapter presents the experimental results of the proposed Swin-KAN framework for IOP prediction based on Scheimpflug images. We evaluate the effectiveness of the data augmentation strategy, quality control mechanism, and multimodal learning through a series of comparative and ablation experiments. Key metrics are reported to assess model accuracy, robustness, and interpretability.

### 5.1. Data Augmentation and Quality Monitoring

To address the challenges of limited sample size and class imbalance in Scheimpflug imaging data, this study proposes a two-stage method that integrates a Cycle-GAN-based data augmentation strategy with a BLS-based quality monitoring mechanism. The overall pipeline consists of two key steps. First, diverse synthetic images are generated using Cycle-GAN to expand the training set required for transfer learning. Second, the BLS is applied to evaluate and filter the augmented images, ensuring that only samples with structural integrity and discriminative validity are used for model training.

**Figure 6:**
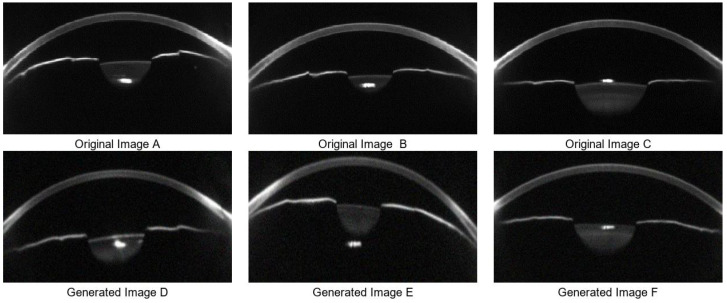
Data Augmentation Results. (**A**–**C**) are three real data sample images, while (**D**–**F**) are corresponding synthetic samples generated by Cycle-GAN-based data augmentation.

In the data augmentation stage, Cycle-GAN is utilized to simulate variations in corneal morphology and anterior chamber structure, thereby enhancing image diversity and coverage of clinical variability. A total of 1200 synthetic images were generated, including 600 samples representing normal IOP and 600 representing elevated IOP. The generated images maintain visual consistency with real samples while introducing key anatomical variations in corneal thickness, anterior chamber depth, and curvature. Representative results are shown in Figure 6. These synthetic images are used for structural feature pre-training of the Swin Transformer, which improves the model’s adaptability and generalization ability in the Scheimpflug feature space.

**Figure 7:**
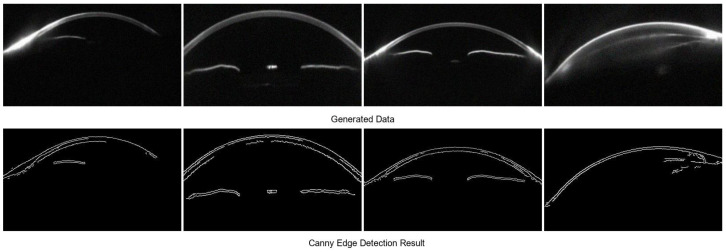
Low-Quality Samples Excluded by the BLS Model.

However, some synthetic samples exhibit structural artifacts and amplified noise. These issues include blurred or discontinuous corneal edges, unrealistic lens reflections, and abnormally bright regions, which interfere with the interpretation of anterior chamber structures and corneal thickness. In addition, large regions of random texture noise can disrupt the learning of anatomical features. To further analyze such artifacts, Canny edge detection is applied to assess the geometric integrity of all synthetic images. The results reveal that low-quality samples often present edge discontinuities, missing contours, or unnatural protrusions in the cornea and chamber angle, which deviate significantly from physiological structures (Figure 7).

To remove such samples, the BLS model is introduced as a quality control module. By leveraging high-dimensional nonlinear feature mapping and pseudoinverse-based regression, BLS quantitatively evaluates image quality. It combines indicators of geometric continuity, texture coherence, and noise level into a unified quality score. Based on both the BLS assessment and Canny detection, all images with structural abnormalities or visible artifacts are excluded, ensuring that only high-quality samples are retained for training.

### 5.2. Comparative Experiments

To validate the performance of the proposed Swin-KAN framework and demonstrate its practical advantages, we conducted a series of comparative experiments. Although no previous studies have directly explored deep learning models for IOP prediction based on Scheimpflug images, we established two categories of baseline models to systematically evaluate the improvements brought by our approach. Specifically, these baselines include: (1) conventional machine learning classifiers based on handcrafted anatomical features, and (2) lightweight CNNs trained on raw image data.

#### 5.2.1. Model Performance Evaluation Metrics

To comprehensively evaluate the performance of each model in the IOP classification task, five widely used classification metrics were adopted. These metrics reflect classification accuracy, model stability, and robustness to class imbalance. Specifically, the evaluation includes Precision, Recall, F1-score, Specificity, and Matthews Correlation Coefficient (MCC), with their mathematical definitions provided in Equations (20)–(24). Each metric captures the model’s capability in identifying positive and negative samples from a different perspective.

(1) Precision: Precision is an important metric for assessing the accuracy of a classification model, especially in classification problems. It measures the proportion of correctly predicted positive samples among all samples predicted as positive.

(20) Pres=TP/(TP+FP)

(2) Recall: Recall evaluates the model’s ability to identify positive samples. It measures the proportion of actual positive samples that are correctly predicted as positive.

(21) Sen=TP/(TP+FN)

(3) F1-score: The F1-score is a comprehensive metric for evaluating classification performance. It combines precision and recall to balance both metrics. The value ranges from 0 to 1, where 0 indicates the model fails to identify positive samples, and 1 represents perfect classification.

(22) F1=2(Pres×Sen)/(Pres+Sen)

(4) Specificity: Specificity is another key metric for evaluating classification models. It measures the proportion of actual negative samples correctly identified as negative. Specificity is particularly important in medical diagnostics to reduce misdiagnosis and avoid unnecessary interventions.

(23) F1=2(Pres×Sen)/Spe=TN/(TN+FP)

(5) Matthews Correlation Coefficient (MCC): MCC is a robust metric for assessing binary classification models. Unlike precision or recall, MCC remains reliable in imbalanced datasets. Its value ranges from −1 to 1, where −1 indicates complete misclassification, 1 represents perfect prediction, and 0 indicates no predictive advantage.

(24) $$MCC=\frac{TP\times TN-FP\times FN}{\sqrt{\left(TP+FP)(TP+FN)(TN+FP)(TN+FN\right)}}$$

Among these metrics, TP represents the number of samples correctly predicted as positive, TN represents the number of samples correctly predicted as negative, FP represents the number of negative samples incorrectly predicted as positive, and FN represents the number of positive samples incorrectly predicted as negative. The introduction of the MCC metric is particularly useful in mitigating the impact of class imbalance in model evaluation. It provides a more reliable performance assessment for medical diagnostic models.

#### 5.2.2. Comparison with Classical Machine Learning Models

To validate the effectiveness of the proposed deep learning framework, several classical machine learning classifiers were constructed and evaluated based on manually extracted ocular diagnostic features. In this experiment, we utilized a series of clinical text-based ocular parameters as features for IOP classification. These parameters include CCT, corneal apex thickness, total corneal volume, anterior chamber depth, anterior chamber volume, and corneal diameter. All features were normalized and concatenated to form a unified feature vector representing each subject’s ocular structure.

Subsequently, to perform binary classification of normal versus elevated IOP, we trained three traditional machine learning models: Support Vector Machine (SVM), Random Forest (RF), and Logistic Regression (LR). To ensure robustness and reduce overfitting, all models were evaluated using a 5-fold cross-validation strategy. Specifically, the training dataset was randomly divided into five equal subsets. In each fold, four subsets were used for training and the remaining one for validation. This process was repeated five times, ensuring that each subset served once as the validation fold. All models were re-trained from scratch in each fold to prevent information leakage, and a fixed random seed was used to ensure reproducibility. The final classification metrics, including accuracy and MCC, were reported as the median values across the five folds to mitigate the effect of outliers. The performance results are summarized in Table 2. Among the three models, SVM achieved the best overall performance, followed by RF, whereas LR exhibited the lowest performance. Nevertheless, all traditional models demonstrated a considerable gap in accuracy and MCC when compared with the proposed Swin-KAN multimodal deep learning framework, which was evaluated separately using the held-out validation and test sets.

This performance gap arises largely because handcrafted or semi-automated feature extraction methods fail to scale effectively and often lead to subjective and inconsistent results in clinical settings. In contrast, the Swin-KAN framework adopts an end-to-end learning paradigm that enables direct extraction and integration of both image-based and structural features from raw data, offering a more robust and interpretable non-contact diagnostic approach.

#### 5.2.3. Comparison with CNN-Based Models

To further validate the effectiveness of the proposed Swin-KAN multimodal framework, we introduced representative CNN models as baseline comparators. By evaluating performance against these well-established architectures, widely used in medical image analysis, we aimed to investigate the advantages conferred by the Swin Transformer backbone and the multimodal fusion strategy. Specifically, we selected three representative CNN models, namely ResNet-50, EfficientNet-B0, and DenseNet121, due to their proven performance, strong feature extraction capabilities, and broad adaptability to transfer learning in medical imaging tasks.

All CNN models were implemented with standard configurations and initialized using pre-trained weights from the ImageNet dataset. To ensure consistency across experiments, Scheimpflug images were resized to 224 × 224 pixels, and the same Cycle-GAN-based augmentation strategy was applied. Training parameters, including batch size, optimizer, and learning rate scheduler, were also kept consistent. Furthermore, the evaluation of CNN models was conducted using the same 5-fold cross-validation strategy described in the previous section. The data splitting, model training process, and metric reporting followed exactly the same protocol as used for the traditional machine learning models to ensure a fair and consistent comparison.

The classification performance of each CNN model is presented in Table 3. Among the models, ResNet-50 achieved the highest F1-score (0.90), followed by DenseNet121 and EfficientNet-B0. Despite their strong generalization capabilities in image classification tasks, all CNN baselines performed worse than the proposed Swin-KAN framework. Notably, traditional CNN models showed lower performance in specificity and MCC, indicating reduced predictive stability when handling imbalanced data.

Compared with other CNN-based models, the Swin-KAN framework shows a relatively longer inference time (35 ms), which is mainly caused by the computational complexity introduced by the hierarchical self-attention mechanism of the Swin Transformer and the multimodal fusion module. However, this increase remains within an acceptable range for practical applications. Given the notable improvements in classification performance, the slightly longer inference time is considered a reasonable trade-off. With the continuous expansion of training datasets and the development of more efficient self-attention mechanisms, future work may explore replacing the current Swin Transformer architecture with lightweight alternatives to further reduce inference time. This would improve the feasibility of deploying such models in real-time clinical environments.

These results suggest that while traditional CNNs are effective in recognizing general visual patterns, they may struggle to capture the complex, fine-grained, and spatially coupled structural features inherent in Scheimpflug images. In contrast, the Swin-KAN framework, by leveraging a Transformer-based architecture and multimodal integration, demonstrates superior capability in extracting deep semantic structures and improving diagnostic robustness.

### 5.3. Grad-CAM Visualization of Corneal Areas

In this study, we explored the visualization method for corneal areas of interest using deep learning models, aiming to uncover potential correlations between these areas and IOP. To confirm the presence of biomarkers in corneal images related to IOP, we applied Gradient-weighted Class Activation Mapping (Grad-CAM) [60] to visualize the model’s predictions from the Swin Transformer. The Grad-CAM technique generates heatmaps that highlight the key regions the model focuses on during decision-making, providing strong support for a deeper understanding of the model’s decision-making mechanism. These visual heatmaps not only reveal the spatial patterns that contribute most to the prediction outcomes but also serve as an intuitive bridge between model inference and clinical reasoning. By correlating highlighted regions with known anatomical or pathological features, clinicians can better interpret the relevance of model predictions, thereby enhancing trust, transparency, and potential diagnostic value in real-world ophthalmic practice.

**Figure 8:**
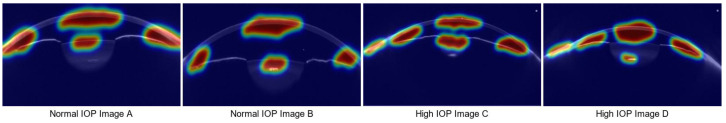
Grad-CAM Visualization of the Swin Transformer Model. (**A**,**B**) show Grad-CAM-generated heatmaps for cases with normal IOP, while (**C**,**D**) illustrate heatmaps for cases predicted as having high IOP.

As shown in Figure 8, the Grad-CAM-generated heatmaps for cases predicted as having ocular hypertension reveal that the model primarily focuses on the corneal region, with particular attention to the CCT and the anterior chamber. This suggests that the model automatically targets anatomical structures that are potentially relevant to clinical assessment during risk prediction.

Although corneal thickness and anterior chamber structures do not directly determine IOP values, previous studies have indicated that these anatomical features can serve as indirect biomarkers of IOP variation. Morphological characteristics of the cornea, such as thickness and curvature, and parameters of the anterior chamber, including depth and volume, may reflect adaptive structural changes resulting from prolonged IOP elevation [61]. For example, increased corneal thickness may result from tissue response to mechanical stress under high IOP. Similarly, reduced anterior chamber volume may indicate impaired aqueous humor dynamics. These findings provide a plausible pathological basis for the model’s focus on these regions [62,63].

To further quantify the association between these anatomical areas and IOP levels, we performed a Pearson correlation analysis between structural imaging features and measured IOP. The results showed a moderate positive correlation between corneal thickness and IOP (*r* = 0.41), suggesting that thicker corneas are generally associated with higher IOP. In contrast, anterior chamber depth and volume were negatively correlated with IOP (*r* = −0.17 and −0.14, respectively), indicating that shallower anterior chambers may be linked to higher risk of ocular hypertension. These findings are consistent with previous reports [64,65,66,67], reinforcing the physiological significance of the model’s attention to these regions.

From the perspective of ocular biomechanics, these observations can be further interpreted. Both the cornea and sclera exhibit hyperelastic properties, with Young’s moduli of 1.5 GPa and 2.25 GPa, respectively, and Poisson’s ratios of 0.49. These tissues respond collectively to fluctuations in IOP [68]. Individual differences in age, tissue elasticity, and aqueous humor dynamics introduce high-dimensional, nonlinear relationships between IOP and structural parameters. Such complexity makes it difficult to model IOP with single variables alone. Deep learning methods, by contrast, offer a more effective diagnostic approach by capturing complex high-dimensional associations between ocular images and IOP, enabling data-driven modeling beyond traditional feature-based techniques.

It is important to note that Grad-CAM works by backpropagating gradients to compute the contribution of each feature map. The resulting heatmaps highlight image regions that contribute most to the model’s prediction for a given class. Therefore, the highly responsive regions in the heatmaps represent structures most relied upon by the model when classifying for ocular hypertension. These highlighted areas not only show what the model “sees,” but may also suggest that certain morphological changes themselves serve as latent predictive markers.

### 5.4. Ablation Experiment and Validation

**Figure 9:**
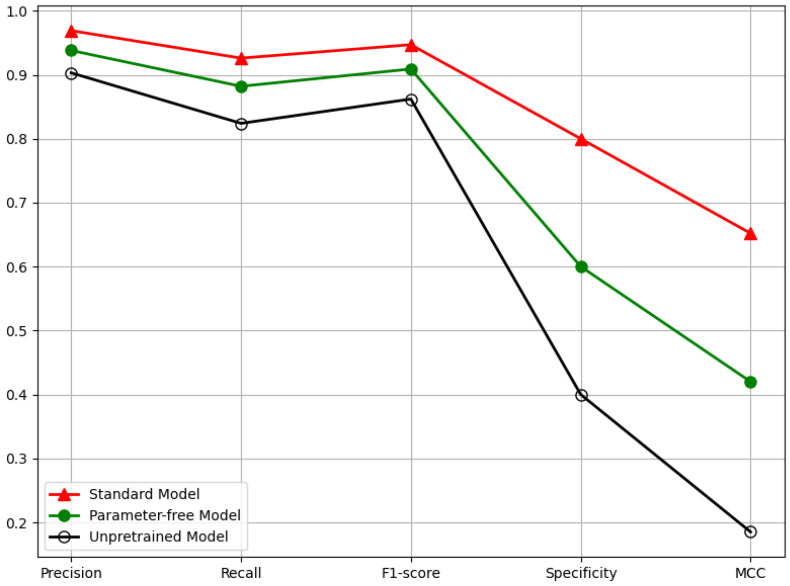
Ablation Experiment Five-Dimensional Performance Evaluation Line Chart for Multimodal-Based High IOP Detection Technology.

To investigate the impact of multimodal data integration and transfer learning on model performance, we evaluated the performance of three models: a single-modal model, a model without pre-training, and a multimodal baseline. As shown in Table 4 and Figure 9, the baseline model trained with both Scheimpflug images and clinical data achieved the best overall performance. Removing clinical information led to noticeable performance degradation, particularly in specificity and MCC, underscoring the importance of medical data in ensuring classification stability. The model without pre-trained weights showed the poorest performance, further highlighting the critical role of transfer learning. These results demonstrate that both pre-training and multimodal data integration are essential for robust and reliable high IOP prediction.

**Figure 10:**
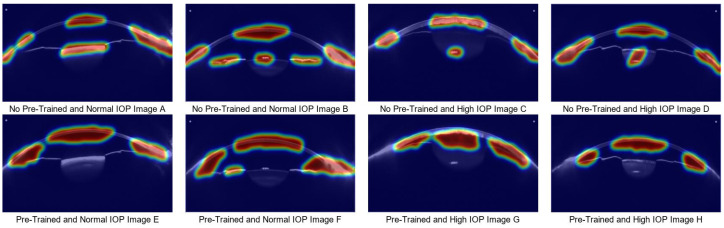
Grad-CAM Comparison Analysis between the Normal Model and the Untrained Model. (**A**–**D**) show heatmaps generated without pretraining: (**A**,**B**) correspond to normal IOP cases, and (**C**,**D**) to ocular hypertension cases. (**E**–**H**) show heatmaps from the pretrained model: (**E**,**F**) correspond to normal IOP cases, and (**G**,**H**) to ocular hypertension cases.)

To further elucidate the differences in model behavior, we employed Grad-CAM to visualize the discriminative image regions that contributed most to the model’s predictions. Figure 10 displays the heatmaps of the baseline model and the model that has not undergone sufficient pre-training. When the model has not been adequately pre-trained, it fails to accurately focus on regions closely related to IOP changes, resulting in decreased stability in high IOP prediction. As shown in Figure 10A–D, the untrained model fails to focus on key areas such as anterior chamber volume or anterior chamber angle, focusing only on the corneal region. This makes the decision-making process in high IOP prediction harder to interpret and understand. In contrast, when the model is sufficiently pre-trained, it can focus on both the anterior chamber and corneal regions, which is crucial for improving the accuracy and transparency of IOP detection. Additionally, when combined with medical numerical data, the model’s predictions become more reliable, thus enhancing its clinical applicability.

In conclusion, this study proposes a multimodal approach that integrates ocular Scheimpflug images with clinical diagnostic data, achieving a fully non-contact method for diagnosing ocular hypertension. Compared with traditional machine learning and single-modality deep learning methods, the proposed approach demonstrates superior classification performance. Moreover, results from ablation studies and Grad-CAM visualizations indicate that the model effectively focuses on ocular regions closely associated with elevated IOP, such as corneal thickness, anterior chamber depth, and anterior chamber angle areas. These findings suggest that the multimodal method based on ocular imaging and clinical diagnostic information holds potential for non-contact detection of ocular hypertension.

## 6. Limitations

This study has two main technical limitations. First, due to medical image data privacy protection policies, the availability of clinical samples from the Scheimpflug imaging system is limited, particularly with a significant class imbalance between normal and abnormal IOP samples. This data scarcity and distribution bias may affect the generalization performance of the classification model. Second, the current mainstream open-source pre-trained models are primarily based on conventional image datasets, which differ significantly from Scheimpflug images in terms of corneal layer features. This discrepancy leads to adaptive bias during model transfer. Furthermore, although data augmentation and transfer learning strategies were employed in this study, there is still room for improvement in feature decoupling under small sample conditions.

## 7. Conclusions

This study proposes a deep learning-based, non-contact IOP detection method that integrates Scheimpflug image data with corneal biomechanical features to predict IOP without the need for physical contact. The research demonstrates that employing Cycle-GAN for data augmentation not only expands the training dataset but also introduces structural variability in corneal morphology, thereby enriching the diversity of training samples. This significantly enhances the robustness and generalizability of the proposed model. In this framework, the Swin Transformer is utilized for efficient extraction of image-based features, while a KAN is adopted for effective multimodal information fusion. By leveraging KAN’s capacity to approximate complex multivariate functions with enhanced interpretability, the model achieves improved performance in high-IOP prediction tasks. Experimental results confirm that the proposed method delivers high precision and stability in detecting elevated IOP levels, highlighting a strong correlation between corneal biomechanical biomarkers and IOP fluctuations. Moreover, Grad-CAM visual analysis reveals meaningful associations between key ocular parameters, such as corneal thickness and anterior chamber depth, and IOP variation, providing physiological insight into the model’s decision-making process.

In future work, we will continue to collect relevant IOP data to enhance the generalizability of the proposed method. Additionally, we plan to incorporate explainability modules and adopt explainable techniques to better elucidate the potential relationship between biomechanical features and IOP variations, thereby improving the transparency of the IOP diagnostic model in clinical decision-making. Finally, we will explore the use of lightweight techniques to refactor the model for deployment in real-world clinical environments.

## Figures and Tables

**Table 1:** Baseline Characteristics of the Study Population.

Parameter	Normal	High IOP
Sample Siz	680	100
Age	67.3	69.5
IOP (mmHg)	15	27
Pupil Center Thickness (mm)	530.5	570.7
Corneal Vertex Thickness (mm)	530.9	572.4
Corneal Volume (mm^3^)	96.1	62.5
Anterior Chamber Depth (mm)	7.9	2.5
Anterior Chamber Volume (${\mathrm{mm}}^{3}$)	115.4	111.7
Corneal Diameter (mm)	2.9	3.1

**Table 2:** Performance comparison between traditional machine learning classifiers and the proposed Swin-KAN model.

Model	Accuracy	Precision	Recall	F1	Specificity	MCC
Ours	0.91	0.97	0.93	0.95	0.80	0.65
SVM	0.72	0.93	0.74	0.82	0.60	0.24
RF	0.67	0.90	0.69	0.78	0.50	0.13
LR	0.63	0.88	0.66	0.77	0.40	0.04

**Table 3:** Performance comparison between CNN-based classifiers and the proposed Swin-KAN model.

Model	Accuracy	Precision	Recall	F1	Specificity	MCC	Inference (ms)
Ours	0.91	0.97	0.93	0.95	0.80	0.65	35
ResNet-50	0.82	0.90	0.90	0.90	0.30	0.20	22
DenseNet121	0.79	0.94	0.81	0.87	0.70	0.39	28
EfficientNet-B0	0.79	0.92	0.84	0.88	0.50	0.28	18

**Table 4:** Ablation experiment analysis and performance comparison table.

Model	Accuracy	Precision	Recall	F1	Specificity	MCC
Ours	0.91	0.97	0.93	0.95	0.80	0.65
Single Modality	0.85	0.94	0.88	0.91	0.60	0.42
No Pretrained	0.77	0.90	0.82	0.86	0.40	0.185

## Data Availability

The data are unavailable for sharing due to privacy.

## Abbreviations

The following abbreviations are used in this manuscript:

IOP	Intraocular Pressure
CNN	Convolutional Neural Networks
BLS	Broad Learning System
KAN	Kolmogorov–Arnold Net
Random Forest	RF
Logistic Regression	LR
